# The Role of Surfactants in Stabilizing Fluorescence Anisotropy for Protein–Aptamer Binding Affinity Measurements

**DOI:** 10.3390/bios15120801

**Published:** 2025-12-06

**Authors:** Bhagya R. Samarakoon, Susan L. Bilderback, Rebecca J. Whelan

**Affiliations:** 1 Department of Chemistry, University of Kansas, Lawrence, KS 66045, USAsusan.bilderback@ku.edu (S.L.B.); 2 Ralph N. Adams Institute for Bioanalytical Chemistry, University of Kansas, Lawrence, KS 66047, USA

**Keywords:** fluorescence anisotropy (FA), aptamer–protein binding affinity, surfactants, Tween 20, Triton X-100

## Abstract

Fluorescence Anisotropy (FA) is a sensitive and efficient technique for quantifying biomolecular interactions, offering advantages such as minimal sample requirements and elimination of separation of bound from unbound species. Thus, it is well suited for aptamer–protein binding affinity studies. However, accurately determining equilibrium dissociation constants (*K_D_*) in FA requires low concentrations of fluorescently labeled aptamers to prevent ligand depletion. A significant challenge arises at low aptamer concentrations due to an unexpected and physically nonmeaningful increase in apparent anisotropy, which impairs accurate data fitting. This anomalous increase in apparent anisotropy may arise from non-specific adsorption of aptamers to surfaces. In this study, we investigated the use of non-ionic surfactants to mitigate these effects and stabilize the anisotropy signal at low aptamer concentrations using the thrombin aptamer as a model system. We evaluated the impact of varying concentrations of two surfactants (Tween 20 and Triton X-100) on plots of anisotropy as a function of aptamer concentration and determined aptamer–protein binding affinities. Addition of 0.1% Tween 20 corrects the anomalous increase in anisotropy at low aptamer concentrations, enabling the use of aptamer concentrations as low as 5 nM in binding assays. Triton X-100 was less effective. By incorporating optimized concentrations of Tween 20, we demonstrated improved assay reproducibility and accuracy in *K_D_* determination, expanding the dynamic range of usable aptamer concentrations in FA-based binding affinity studies. Similar benefits were observed with the clinically relevant aptamer s10yh2 and human serum albumin. These findings provide a practical strategy for enhancing the robustness of FA measurements and may be applicable to other aptamer–target systems and high-throughput assay formats.

## 1. Introduction

Fluorescence Anisotropy (FA) is widely used to study solution-phase biomolecular interactions. This method has found broad application in the study of protein–ligand [1,2,3], DNA–protein [4], RNA–protein [5], and aptamer–protein binding due to its high sensitivity, minimal sample requirements, and homogenous assay format [6,7,8]. Compared to other techniques used for binding affinity studies, FA offers many advantages for protein–aptamer binding studies. This technique does not require the separation of bound from unbound species, which minimizes sample loss and experimental artifacts. High sensitivity and low sample volume requirements make FA an ideal method for studying biomolecular interactions with limited samples [9,10,11]. Unlike surface-based techniques such as Surface Plasmon Resonance (SPR) [1,12,13] and Enzyme Linked Immunosorbent Assay (ELISA) [14,15,16], FA does not require ligand immobilization, which can introduce artifacts or disrupt weak or transient interactions. Instead, FA allows binding events to be monitored under native conditions, which is advantageous for studying low-affinity interactions or labile complexes and compatible with high-throughput screening [17,18,19]. FA experiment designs are flexible and compatible with a variety of experimental conditions including varying ionic strengths, pH levels, and buffer compositions, unlike electrophoresis-based techniques. This flexibility enables FA to be used in a variety of applications for a broad spectrum of experimental needs [7,20,21].

**Figure 1:**
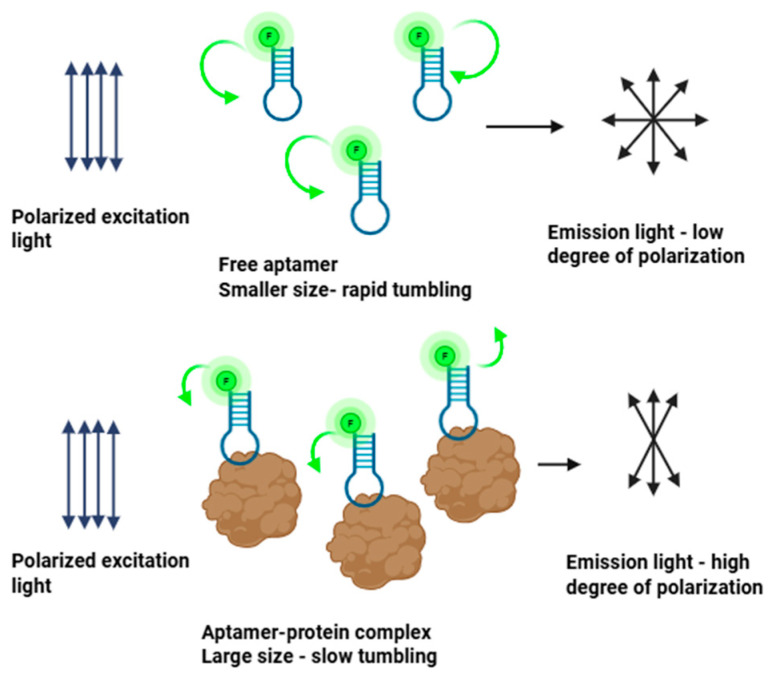
Principle of fluorescence anisotropy binding assays. The green arrows illustrate the rate of tumbling, which is faster for free aptamer (longer arrow, upper) and slower for aptamer–protein complex (shorter arrow, lower). Image created with Biorender.com (accessed on 21 August 2025).

The principle of FA is based on the rotational diffusion of fluorescently labeled molecules. When excited with plane-polarized light, a fluorophore emits light that retains a degree of polarization depending on the extent to which the molecule has rotated during its residence in the excited state. While small molecules tumble rapidly in solution, leading to depolarized emission and low anisotropy, a fluorescent molecule bound to a larger partner such as a protein, nanostructure, or vesicle experiences a decrease in rotational diffusion rate. The emitted fluorescence retains more of its original polarization, resulting in a higher anisotropy signal. This size-dependent modulation of anisotropy enables FA to serve as a direct quantitative readout of binding interactions in solution [8,22]. The principle of FA binding assays is illustrated in Figure 1.

FA is calculated from the ratio of the emitted fluorescent intensities parallel (I_‖_) and perpendicular (I_┴_) to the excitation plane, as shown in Equation (1) [4,23].

(1) $$r=\frac{I_{\|}-I_{┴}}{I_{\|}+2I_{┴}}$$

Anisotropy, described by the unitless parameter *r*, ranges from 0 to 0.4 under ideal conditions. Anisotropy changes are sensitive to molecular mass changes as small as kilodaltons, enabling discrimination between free and bound fluorescent ligands with minimal reagent consumption. Moreover, FA is compatible with a variety of fluorophores and optical plate reader configurations and can be performed in microplates with volumes as low as 10 μL, further supporting its utility for high-throughput applications and in resource-limited settings.

Aptamers are small single-stranded oligonucleotides that can fold into specific secondary structures that bind to target ligands with high affinity and specificity [16,24,25]. Quantifying the equilibrium dissociation constant (*K_D_*) that describes the binding affinity between a fluorescently labeled aptamer and its protein target involves titrating the protein across a range of concentrations and measuring the resulting change in anisotropy [5,26,27,28,29,30,31]. These data are typically fit to a Langmuir binding isotherm, which assumes a simple 1:1 binding interaction in which free aptamer and free protein are in equilibrium with an aptamer–protein complex (Equation (2)).

(2) $$f_{b}=\frac{\left.[P\right]}{\left.K_{D}+[P\right]}$$

where *f*_b_ is the fraction bound, [P] is the protein concentration, and *K_D_* is the equilibrium dissociation constant of the complex. A critical assumption of this model is that the concentration of free protein [P] is effectively equal to the total protein added, [P]_t_. This assumption holds true only when the concentration of the aptamer [A]_t_ is significantly lower than *K_D_*, such that ligand depletion is negligible. If aptamer concentration approaches or exceeds *K_D_*, a significant fraction of protein maybe sequestered into complex [PA], causing [P] << [P]_t_, leading to deviation from Langmuir behavior. This distortion effects the apparent binding curve, reduces fitting accuracy, and results in errors in *K_D_* estimations unless a quadratic or mass balance corrected binding model is used [1,32].

The equilibrium relationship for a reversible 1:1 binding is given by [A]_t_ = [A]_f_ + [PA], [P]_t_ = [P]_f_ + [PA] and the binding constant is defined by Equation (3):

(3) $$K_{D}=\frac{{\left[A\right]}_{f}{\left[P\right]}_{f}}{\left[PA\right]}$$

when aptamer concentration is kept low, [A]_f_ ≈ [A]_t_ and [P]_f_ ≈ [P]_t_ and the Langmuir approximation (Equation (2)) is valid. This highlights the importance of operating in a ligand-limiting regime, typically maintaining aptamer concentrations lower than the expected *K_D_*, especially when working with nanomolar or sub-nanomolar affinities.

Despite this theoretical requirement, a recurring experimental challenge in FA assays is the unexpected increase in anisotropy observed at low aptamer concentrations, even in the absence of the target protein. This phenomenon introduces noise in the lower region of the binding curve, impairs signal discrimination between bound and unbound states, and ultimately limits the usable concentration range of the aptamer. In practice, this has led researchers to empirically select aptamer concentrations at or just below the binding curve plateau, compromising optimal fitting conditions and potentially affecting the validity of derived *K_D_* values [21,27,33].

We hypothesized that observed anomalous increases in anisotropy at low aptamer concentrations stem from non-specific adsorption of aptamers to hydrophobic well surfaces, or intermolecular aggregation driven by hydrophobic interactions in solution. Such artifacts are especially prominent in low-volume assays using 384 microwell plates, where the surface area-to-volume ratio is high and aptamer adsorption can disproportionately affect free aptamer concentration. This adsorption to the microplate surface can restrict the rotational freedom of the fluorophore, mimicking the slower tumbling expected of a bound complex and artificially increasing anisotropy values.

One strategy to mitigate non-specific binding is adding non-ionic surfactants to the assay buffer. Surfactants such as Tween 20 and Triton X-100 are molecules composed of hydrophilic polyethylene glycol (PEG) chains and hydrophobic alkyl groups allowing them to adsorb to hydrophobic surfaces and form protective layers that block non-specific interactions [34]. The hydrophobic region consists of alkyl chains with 8 to 22 carbons, while the hydrophilic region consists of polar moieties. The presence of two such opposite functions in molecules is called an amphiphilic molecular structure [35,36]. However, despite their frequent use in FA assays, the effect of different concentrations and types of surfactants on the FA signal, particularly under low ligand conditions, has not been systematically studied in the context of aptamer–protein binding studies.

In addition to surfactants, PEG-passivation is another widely used approach to minimize non-specific adsorption on assay surfaces, including well plates [37,38,39]. Though effective, these surface modification methods require pre-treatment of the well plates through coating or chemical activation. PEG can instead be conveniently added as a soluble reagent to assay mixtures in the form of Tween 20 or Triton X-100, providing an easy and effective way to reduce nonspecific adsorption. This strategy offers a practical alternative that maintains assay simplicity and improves signal specificity, provided that the interaction of interest is not perturbed.

In this study, we have incorporated different concentrations of two non-ionic surfactants, Tween 20 and Triton X-100, into samples containing thrombin and fluorescently labeled thrombin-binding aptamers. Thrombin–aptamer binding is a well-characterized model system with nanomolar affinity. We used high-efficiency 384 low-volume well plates with low sample volumes which enables improved performance in FA experiments [40]. We found that the addition of Tween 20 successfully removed the anomalous anisotropy increase at low aptamer concentrations, thereby enabling the use of lower aptamer concentrations in FA binding experiments. We further demonstrate the effect of surfactant using the clinically relevant aptamer s10yh2 and its binding towards human serum albumin (HSA).

## 2. Materials and Methods

### 2.1. Reagents and Materials

All aptamers were purchased from Integrated DNA Technologies (Coralville, IA, USA). These included Texas Red (TR) and fluorescein (FAM) labeled thrombin-binding 15mer [41], thrombin-binding 29mer [42], and ITGB3-binding s10yh2 [43]. The sequence of the thrombin-binding 15mer was 5′-GGT TGG TGT GGT TGG-3′. The sequence of the thrombin-binding 29mer was 5′-AGT CCG TGG TAG GGC AGG TTG GGG TGA CT-3′. The sequence of s10yh2 was 5′-TCC ACG ATG TAG GAT CCA CAT GAG CGC TCC TGT TAC CCT G-3′. Aptamers were purified by the vendor using standard desalting and obtained as lyophilized solids. Aptamers were reconstituted in TE buffer (10 mM Tris, 0.1 mM EDTA, pH 8) to obtain a 100 μM solution. TGK buffer (192 mM Tris, 5 mM KH_2_PO_4_, 25 mM glycine hydrochloride) was used as the binding buffer. Thrombin from human plasma, bovine serum albumin (BSA), and human serum albumin (HSA) were purchased from Sigma Aldrich (St. Louis, MO, USA). Thrombin was reconstituted in a solution of 1 mg/mL BSA in Milli-Q water to obtain a 1000 U/mL solution. 384-well low-volume round-bottom, standard opaque well plates were purchased from Sigma Aldrich. Fluorescence anisotropy assays were performed in a SpectraMax M5 plate reader from Molecular Devices (San Jose, CA, USA). For thermal cycling of aptamers, an Eppendorf Mastercycler Nexus Gradient thermocycler was used. Surfactants Triton X-100 and Tween 20 were purchased from Fisher Scientific (Waltham, MA, USA) and used without further purification.

### 2.2. Experimental Methods

#### 2.2.1. Plots of Anisotropy as a Function of Aptamer Concentration for TR- and FAM-Labeled Thrombin-Binding 29mer

A working stock solution of 400 nM was prepared from TR-labeled thrombin-binding 29mer. Aptamer samples were heat cycled at 95 °C for 3 min and immediately cooled to 4 °C to refold the aptamer. Aptamer dilutions of 0, 10, 25, 50, 100, 200, 250, 300, 400 nM were prepared in TGK buffer. 10 μL of each aptamer dilution was loaded into 384 low-volume standard opaque well plates and the resulting anisotropies and fluorescence intensities were measured using a Spectramax M5 plate reader with excitation at 595 nm, emission detected at 615 nm, and a wavelength cut-off at 610 nm. For FAM-labeled aptamers, a similar dilution scheme was applied, and the excitation and emission wavelengths were 485 nm and 525 nm, respectively, with a wavelength cut-off at 515 nm.

#### 2.2.2. Binding Affinity of Thrombin and TR-Labeled Thrombin-Binding 29mer

Thrombin protein reconstituted in a solution of 1 mg/mL BSA in Milli-Q water was used to prepare dilutions ranging from 0 to 1000 U/mL. Heat-cycled TR-labeled thrombin-binding 29mer dilutions were prepared in TGK with or without surfactants. Refolded aptamer was added to each protein dilution and 10 μL of each protein–aptamer mixture was loaded into wells in triplicate. Plates were sealed and incubated in the dark at 25 °C for 40 min to allow the sample to equilibrate. Change in anisotropy was plotted as a function of thrombin concentration and fitted to the Hill equation using Igor-Pro 8.04 software (Wavemetrics, Inc., Portland, OR, USA). Although the Langmuir binding isotherm, which assumes a simple 1:1 interaction, is widely used, alternative binding models such as the Hill equation can be used in systems that show cooperativity (*n*) or multiple binding sites. Here, we used the Hill equation to fit thrombin/aptamer binding data because we have observed that the thrombin aptamer complex shows positive cooperative binding in both FA assays and in affinity probe capillary electrophoresis binding assays [32]. The argument made in the Introduction about the importance of avoiding ligand depletion pertains to binding systems that follow the Hill equation (with cooperative binding) as well as to binding systems that follow Langmuir (non-cooperative) binding.

#### 2.2.3. Incorporating Different Concentrations of Tween 20 and Triton X-100 into Binding Buffer

To evaluate the effect of surfactants, anisotropy as a function of aptamer concentration was measured for TR-labeled thrombin-binding 29mer in TGK buffer containing Tween 20 or Triton X-100 at concentrations of 0.01%, 0.05%, and 0.1% (*v*/*v*). The background-subtracted values were used to construct plots of anisotropy as a function of aptamer concentration and identify any concentration-dependent anomalies.

#### 2.2.4. Comparison of Anisotropy vs. Concentration for Different Fluorophores and Aptamer Lengths with and Without 0.1% Tween 20

Anisotropy was measured as a function of aptamer concentration for TR 29mer, TR 15mer, and FAM 15mer to assess the effect of surfactants at different aptamer lengths and with different fluorophores. To analyze the effect of surfactants, the trend in anisotropy as a function of aptamer concentration was determined in the absence and presence of 0.1% Tween 20. The resulting anisotropy signal was measured at the relevant excitation and emission wavelengths.

#### 2.2.5. Application of Surfactant Modification to Clinically Relevant Aptamers

To assess the generalizability of surfactant optimization beyond thrombin, the same FA assay workflow was applied to the binding of TR-labeled s10yh2 aptamer (originally reported as an aptamer targeting integrin beta 3 [43]) to HSA. The binding assay was conducted in PBS buffer with 5 mM MgCl_2_ containing 0.05% Tween 20 using a fixed aptamer concentration of 75 nM. After equilibration FA measurements were performed as described above.

## 3. Results and Discussion

### 3.1. Analysis of Anisotropy as a Function of Aptamer Concentration

**Figure 2:**
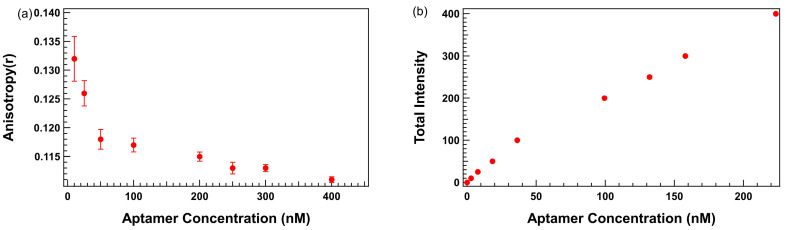
Trends in fluorescence anisotropy and fluorescence intensity as a function of TR-labeled thrombin-binding 29mer aptamer concentration. (**a**) Anisotropy (y-axis) vs. aptamer concentration (x-axis). (**b**) Fluorescence intensity (y-axis) vs. aptamer concentration (x-axis). Error bars represent the standard error of the mean (SEM). Each data point represents 3 biological replicates (independent wells) each measured in triplicate, totaling n = 9 technical replicates.

Plots of anisotropy as a function of aptamer concentration obtained in TGK buffer without surfactants revealed a reproducible increase in anisotropy at low aptamer concentrations, particularly in the 10–50 nM range, as shown in Figure 2a. This upward deviation from the anticipated horizontal line with slope = 0 was not observed in fluorescence intensity readings, which increased linearly across all concentrations as shown in Figure 2b, confirming accurate pipetting and aptamer quantitation. The linear increase in fluorescence intensity with concentration suggests that the observed anisotropy anomaly is not a result of fluorescence concentration artifacts, but likely due to non-specific interactions between the aptamer and the assay environment.

Several hypotheses may be proposed to explain this phenomenon, including aptamer adsorption to the polystyrene surface of the microplate wells and the formation of transient non-covalent aggregates. These interactions may restrict the rotational freedom of the fluorescently labeled aptamer, artificially increasing anisotropy even in the absence of binding. This finding presents a significant barrier to the use of low aptamer concentrations, which are otherwise essential for avoiding ligand depletion and enabling accurate *K_D_* estimation under Langmuir assumptions.

### 3.2. Thrombin-Aptamer Binding in the Absence of Surfactant

Based on the plot of anisotropy as a function of aptamer concentration in the absence of surfactants, 75 nM was the lowest aptamer concentration that could be reliably used without encountering baseline deviation. Below this concentration, the anisotropy signal deviated from linearity and entered a region where surface interactions or hydrophobic effects artificially inflated the signal. Above 75 nM, the measured anisotropy behaves as expected, being invariant with increasing aptamer concentration.

**Figure 3:**
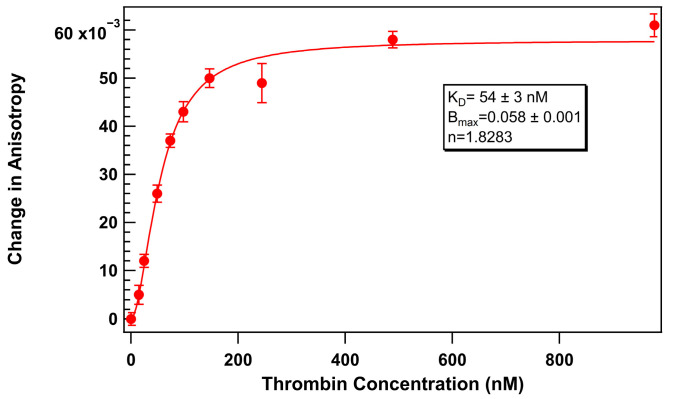
Change in anisotropy of TR-labeled thrombin-binding 29mer (75 nM) upon addition of thrombin without added surfactant. Error bars represent the standard error of the mean (SEM). Each data point represents 3 biological replicates (independent wells) each measured in triplicate, totaling n = 9 technical replicates.

Therefore, to establish a baseline comparison, we first assessed the thrombin aptamer binding curve under surfactant-free conditions using 75 nM of thermally refolded TR-labeled thrombin-binding 29mer aptamer. Thrombin protein was titrated with a range of 0 to 1000 U/mL thrombin in an aqueous solution containing 1 mg/mL BSA. FA measurements were recorded after 40 min of equilibration at 25 °C. The resulting binding curve (Figure 3) displayed a characteristic shape indicative of a specific interaction between 29mer and thrombin with a *K_D_* of 54 ± 3 nM.

### 3.3. Effects of Surfactants

Previous studies have reported the incorporation of surfactants into FA experiments in different concentrations [44,45,46]. For example, Li et al. incorporated 0.1% Tween 20 in an FA-based assay where an aptamer labeled with Lissamine Rhodamine B was used as a label for Ochratoxin A, reporting improved sensitivity of the assay. The study also suggests that Tween 20 had a negligible effect on the FA value of the affinity complex of the aptamer and the fluorescent probe [47]. Zhai et al. optimized a high-throughput FA assay for screening Bfl-1 inhibitors, demonstrating how assay conditions, including surfactants, influence FA measurements. These findings highlight the need to account for surfactant effects in FA assays to ensure accurate interpretation of molecular interactions [19]. Recent studies also suggest that low concentrations (~0.01%) of surfactants such as Triton X-100 and Tween 20 can minimize hydrophobic interactions and reduce protein adsorption to well surfaces [17]. Mohommad et al. developed a fluorescence polarization (FP; analogous to FA) assay for high-throughput screening of inhibitors against HIV-1 Nef-mediated CD4 down-regulation. They optimized the assay through the addition of surfactants Triton X-100 and Tween 20 at varying concentrations and reported that 0.01% Triton X-100 was most effective at reducing protein aggregation [48]. However, a systematic investigation of the effects of varying Tween 20 and Triton X-100 remains unexplored. Given the widespread applications of FA in studying biomolecular interactions, further investigation into the optimal surfactant conditions is essential for improving assay reliability and accuracy.

**Figure 4:**
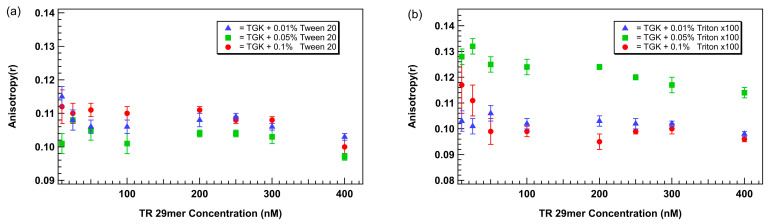
Plot of anisotropy as a function of aptamer concentration with the addition of different concentrations of surfactants (**a**) Tween 20 and (**b**) Triton X-100. Aptamer is a TR-labeled thrombin-binding 29mer. Error bars represent the standard error of the mean (SEM). Each data point represents 3 biological replicates (independent wells) each measured in triplicate, totaling n = 9 technical replicates.

Thus, to mitigate the unexpected anisotropy elevation, we evaluated the effects of two non-ionic surfactants, Tween 20 and Triton X-100, at concentrations of 0.01%, 0.05% and 0.1% (*v*/*v*). In TGK buffer supplemented with Tween 20, a dose-dependent flattening of the anisotropy baseline was observed. Notably, 0.1% Tween 20 eliminated the low-concentration anisotropy increase, producing a flat and reproducible baseline across 0–75 nM aptamer concentrations (Figure 4a). Similar trends were observed for Triton X-100, but with less effective suppression of the anomaly at equivalent concentrations. At 0.01% Triton X-100 had minimal impact, while 0.05% produced partial stabilization. Samples containing 0.1% Triton X-100 exhibited higher baseline variability compared to Tween 20 (Figure 4b).

These results support the conclusion that nonionic surfactants reduce non-specific interactions responsible for spurious anisotropy increases. Tween 20, a milder surfactant with smaller micelle-forming tendencies than Triton X-100, appears better suited to stabilizing aptamer behavior in low-volume FA assays.

Surfactants such as Tween 20 and Triton X-100 mitigate non-specific adsorption primarily by coating hydrophobic surface regions that can attract nucleic acids and proteins through weak hydrophobic and electrostatic interactions. Tween 20 is composed of a polyoxyethylene sorbiton ester, which forms a hydrated PEG layer that reduces DNA adsorption by creating a steric and hydration barrier while preventing DNA strands from interacting with exposed hydrophobic areas on the plate surface [49]. Triton X-100 contains an aromatic hydrophobic core and shorter ethylene oxide chains, which provide a stronger solubilizing capacity [50]. We therefore chose 0.1% Tween 20 as our optimum surfactant concentration for this assay since its gentler and non-denaturing properties help maintain aptamer–protein complex integrity while effectively minimizing aptamer surface adsorption to the well surface.

While surfactants generally improve assay performance by minimizing non-specific adsorption, their effects are concentration-dependent and can become detrimental under certain conditions. At higher concentrations, surfactants like Tween 20 and Triton X-100 can perturb protein tertiary structure, alter aptamer folding, and mask binding interfaces [50]. These effects result in reduced affinity or changes in fluorescence anisotropy signals. Triton X-100 in particular has a stronger detergent activity due to its aromatic hydrophobic core, which can disrupt hydrophobic interactions critical for protein stability and aptamer protein conformational recognition. Excessive Tween 20 can also interfere with electrostatic interactions and displace weakly bound ligands from hydrophobic sites. To avoid these effects, the surfactant concentration in this study was maintained at a low, optimized level.

### 3.4. Comparison of Plots of Anisotropy as a Function of Aptamer Concentration for Different Fluorophores and Aptamer Lengths with and Without 0.1% Tween 20

**Figure 5:**
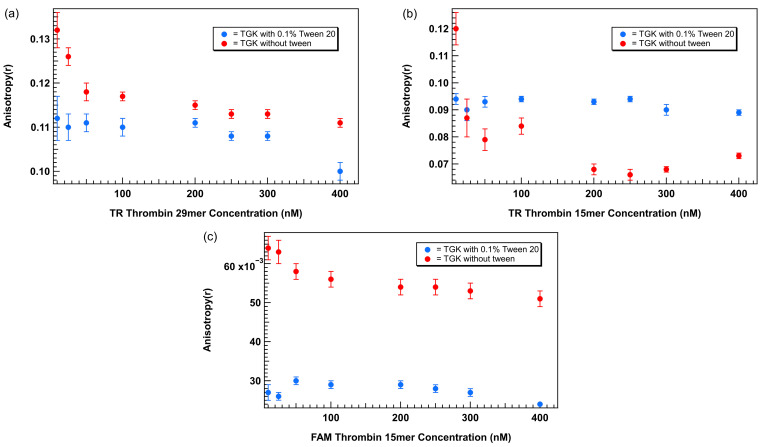
Plots of anisotropy as a function of aptamer concentration with and without 0.1% Tween 20 for aptamers with different lengths and fluorophores. (**a**) TR-labeled thrombin-binding 29mer, (**b**) TR-labeled thrombin-binding 15mer, (**c**) FAM-labeled thrombin-binding 15mer. Error bars represent the standard error of the mean (SEM). Each data point represents 3 biological replicates (independent wells) each measured in triplicate, totaling n = 9 technical replicates.

To directly assess the impact of Tween 20 on FA measurements, we compared plots of anisotropy as a function of aptamer concentration generated for three aptamer–fluorophore combinations: TR-labeled 29mer, TR-labeled 15mer, and FAM-labeled 15mer thrombin-binding aptamers in the presence and absence of 0.1% Tween 20 in TGK buffer. Anisotropy values were measured across a concentration range of 0 to 400 nM for each aptamer type and plotted as a function of aptamer concentration. The results are shown in Figure 5. In the absence of Tween 20 the TR-labeled 29mer (Figure 5a) showed a distinct increase in anisotropy at lower concentrations (10–50 nM), with values peaking above 0.13 before gradually decreasing and stabilizing by ~75 nM. This unexpected elevation at low concentrations introduces artifacts in the baseline, complicating the use of ligand-limiting concentrations critical for accurate binding affinity analysis. The addition of 0.1% Tween 20 effectively flattened the curve across the full concentration range. In Tween 20-containing buffer, the baseline remained stable, enabling the use of concentrations below 75 nM without the risk of overestimating the unbound anisotropy signal.

A similar trend was observed with the TR-labeled 15mer (Figure 5b). In TGK buffer without Tween 20, anisotropy increased sharply at concentrations below 75 nM, peaking at ~0.12 before dropping at higher concentrations. In contrast, the curve generated in 0.1% Tween 20 maintained a consistent anisotropy baseline between 0.08 and 0.09 across the concentration range, with no low-concentration artifact. These results confirm that surfactants’ stabilizing effect is not sequence- or length-specific, but generalizable across different thrombin aptamer formats.

The FAM-labeled 15mer showed the most pronounced baseline instability in the absence of Tween 20. Anisotropy value reached >0.06 at lower concentrations and decreased toward 0.05 at higher concentrations. In the presence of 0.1% Tween 20, anisotropy values remained consistently ~0.03 across the full concentration range, with minimal variability. The larger discrepancy observed between the two conditions for the FAM-labeled aptamer suggests that the nature of the fluorophore may also influence the FA signal. According to the literature, the FAM label undergoes motion independent of the aptamer due to its negative charge. This introduces more variability for FAM in FA experiments compared to the TR label, making FAM non-ideal for FA experiments [28]. Additionally, FAM and its derivatives such as FITC have a smaller Stokes shift (23 nM), which makes the fluorophore more sensitive to light scattering and background intensity [21].

Across all three labeled aptamer systems, the addition of 0.1% Tween 20 eliminated the low concentration elevation in anisotropy observed in surfactant-free conditions. This non-specific signal increase is likely due to aptamer adsorption to polystyrene surfaces or hydrophobic interactions, both of which restrict rotational diffusion and artificially inflate the anisotropy readings. Tween 20, a non-ionic surfactant with amphiphilic properties likely coats the well surface and minimizes these interactions by reducing surface tension and hydrophobic binding. This supports its general use as a low-cost, non-interfering additive in FA assays particularly when working with nanomolar aptamer concentrations.

### 3.5. Using Surfactants in Thrombin Aptamer Binding Assays

**Figure 6:**
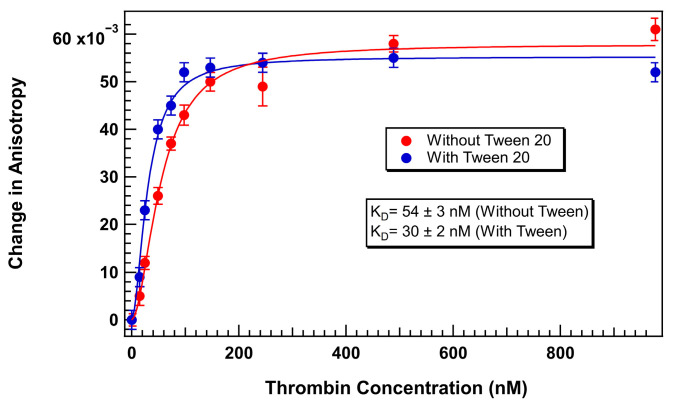
TR-labeled thrombin 29mer binding curve with and without Tween 20 (Aptamer concentration = 75 nM). Error bars represent the standard error of the mean (SEM). Each data point represents 3 biological replicates (independent wells) each measured in triplicate, totaling n = 9 technical replicates.

Following optimization, thrombin-aptamer binding assays were repeated using 75 nM TR-labeled 29mer aptamer in TGK buffer supplemented with 0.1% Tween 20. A well-defined sigmoidal binding curve was obtained with a wider dynamic range and improved curve fitting, as shown in Figure 6. The *K_D_* calculated under these optimized conditions was 30 ± 2 nM, demonstrating both higher binding affinity and improved precision over the surfactant-free condition. Inclusion of Tween 20 did not alter the apparent binding affinity but reduced the standard errors in each replicate reading.

### 3.6. Thrombin and TR-Labeled 29mer FA Assay with Lower Aptamer Concentrations

Following the successful stabilization of anisotropy as a function of aptamer concentration using 0.1% Tween 20, we next evaluated whether lower aptamer concentrations could be reliably used in binding assays, an important requirement for maintaining ligand-limiting conditions necessary for accurate *K_D_* estimation based on the Langmuir binding model. Specifically, we performed thrombin titration experiments using TR-labeled 29mer at concentrations of 50 nM, 25 nM, and 5 nM in TGK buffer supplemented with 0.1% Tween 20.

**Figure 7:**
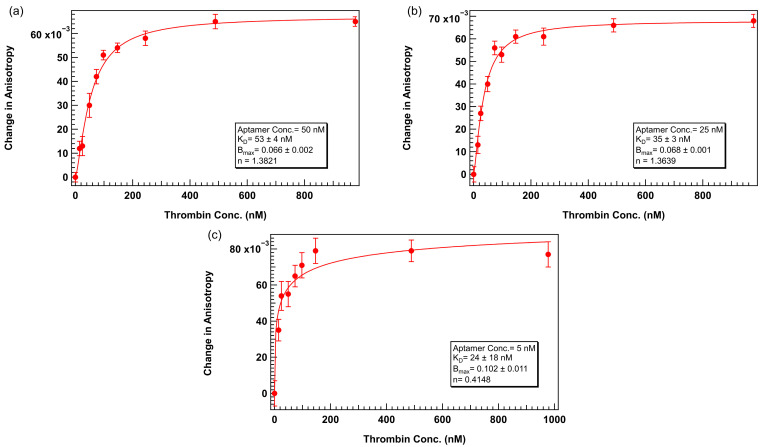
Thrombin 29mer binding assay with lower aptamer concentrations. Binding buffer = TGK + 0.1% Tween 20. Within each assay, aptamer concentration was fixed at (**a**) 50 nM, (**b**) 25 nM, or (**c**) 5 nM. Thrombin concentration varied. Error bars represent the standard error of the mean (SEM). Each data point represents 3 biological replicates (independent wells) each measured in triplicate, totaling n = 9 technical replicates. The solid lines represent binding curves calculated assuming positive cooperativity (Hill equation).

Binding curves obtained under these lower aptamer concentrations showed well-defined sigmoidal behavior across the thrombin concentration range while maintaining the *K_D_* value within a factor of 2, as shown in Figure 7. This observation confirmed that lower aptamer concentrations still provided sufficient signal change for reliable binding analysis. The *K_D_* value for 50 nM (Figure 7a) aptamer concentration was 53 ± 4 nM and the 25 nM (Figure 7b) dataset resulted in a K_D_ of 35 ± 3 nM, both in reasonable agreement with the affinity determined using 75 nM aptamer. The agreement in *K_D_* values across concentrations confirms that ligand depletion effects were minimal and the assay remained within the valid dynamic range. These results demonstrate that inclusion of Tween 20 not only improves signal stability but also enables the use of lower aptamer concentrations without introducing baseline artifacts or compromising binding curve quality. This expands the flexibility of FA assay design, particularly for applications involving scarce or expensive ligands and ensures theoretical consistency with kinetic binding models that assume negligible ligand depletion.

At the lowest tested concentration of 5 nM (Figure 7c) although a weak binding trend could still be discerned, the small change in anisotropy and reduced total fluorescence intensity at this concentration limited the reliability of curve fitting. The calculated *K_D_* for this dataset showed a higher degree of error and less confidence in model fitting, suggesting that instrument sensitivity and signal-to-noise ratio become limiting factors at this ultra-low aptamer regime, even under surfactant-optimized conditions.

The *K_D_* values reported here for the 29mer–thrombin system lie within the range of values previously reported in the literature, tabulated in Mears et al. [32]. Reported *K_D_* values for the 29mer–thrombin range from a minimum of 0.5 nM to a maximum of 255 nM, with differences attributable to the method used to measure binding and the buffer conditions. In comparable experiments [32] that we performed using fluorescence anisotropy to characterize the binding of Texas Red-labeled 29mer aptamers to thrombin, we found the *K_D_* value to be 45.6 ± 2.5 nM, in good agreement with the values reported here.

### 3.7. Validating the Effects of Surfactants in FA Measurements for Clinically Relevant Aptamers

To evaluate the effect of surfactants in a clinically relevant aptamer–protein complex binding measurement, we compared the performance of TR-labeled s10yh2 aptamer titrated against HSA in PBS-M buffer with and without Tween 20.

**Figure 8:**
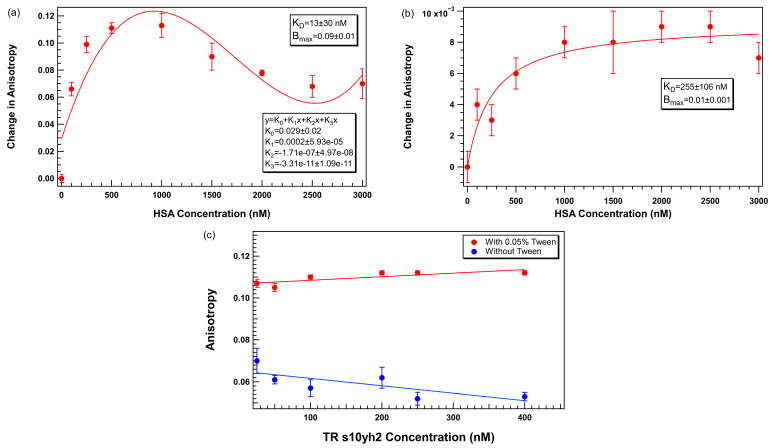
TR-labeled s10yh2 aptamer binding to HSA in PBS-M buffer (**a**) without Tween 20 and (**b**) with 0.05% Tween 20, (**c**) plot of TR-labeled s10yh2 anisotropy as a function of aptamer concentration with and without added Tween 20. Error bars represent the standard error of the mean (SEM). Each data point represents 3 biological replicates (independent wells) each measured in triplicate, totaling n = 9 technical replicates. The solid lines in a) and b) represent binding curves calculated by Igor Pro assuming a 1:1 binding model. The solid lines in c) are linear fits to the data.

In the absence of Tween 20 (Figure 8a), the anisotropy signal increased with increasing HSA concentrations up to ~1000 nM, reaching a maximum change in anisotropy. Beyond this concentration, the binding response plateaued and slightly decreased at higher HSA levels. This trend shows saturable binding between the aptamer and HSA with potential nonspecific effects becoming more pronounced at elevated protein concentrations. Notably, a 1:1 binding model fits the data poorly, and a non-standard function is required to model the data well; a four-parameter polynomial fit function is shown in 8a.

When 0.05% Tween 20 was included in the buffer (Figure 8b), it yielded a more consistent binding profile across the full HSA concentration range. While the absolute magnitude of anisotropy change was reduced (maximum change ~ 10^−3^), the data were smoother, with no evidence of signal decline at higher protein concentrations, and the 1:1 binding model is a better fit to the experimental data.

Figure 8c shows plots of anisotropy as a function of aptamer concentration for TR-labeled s10yh2 in the presence and absence of 0.05% Tween 20. It shows that in the absence of Tween 20, the anisotropy increases at low aptamer concentrations and shows greater variability across concentrations. This effect can be quantified by fitting linear functions to the data. The best-fit line for the data with Tween 20 (red circles) has a slope of 1.7 ± 0.6 × 10^−5^, which is closer to 0 than for the data without Tween 20 (blue circles) for which the slope is −3.5 ± 1.6 × 10^−5^. In addition, in the presence of 0.05% Tween 20, the anisotropy values were markedly higher and stable across the full concentration range, with smaller root mean square deviation (calculated by Igor Pro as the standard deviation of the residuals; 6.3 × 10^−6^ with Tween 20 and 1.6 × 10^−5^ without Tween 20).

These data indicate that Tween 20 minimizes non-specific interactions, preventing surface adsorption while improving data quality. Although the dynamic range of anisotropy is smaller in the presence of Tween 20, the improved stability and reduced non-specific binding interferences make the measurements more reliable. For clinical and translational applications, it is critical to have robust and reproducible measurements, where incorporating Tween 20 into FA assays provides clear advantages. The observed reduction in dynamic range may be caused by mild surfactant-protein interactions as well as improved surface passivation. Lower Tween 20 concentrations (for example, 0.02%) may offer a desirable balance between minimizing adsorption and minimizing protein perturbation. Optimizing surfactant levels for each aptamer–protein system is recommended.

## 4. Conclusions

This study demonstrates that the addition of non-ionic surfactants provides an effective and generalizable solution to a limitation in FA-based aptamer–protein binding assays. We showed that unexpected anisotropy that increases at low aptamer concentrations, which have previously restricted the use of ligand-limiting conditions necessary for accurate dissociation constant (*K_D_*) determinations, arise from aptamer adsorption to microplate surfaces or nonspecific interactions. Systematic evaluation of surfactants revealed that Tween 20 consistently eliminated these artifacts across multiple aptamer sequences and fluorophores, including both TR and FAM labels.

Incorporating Tween 20 eliminated the unexpected and physically nonmeaningful increase in anisotropy at low aptamer concentration and enabled the use of lower aptamer concentrations (down to 25 nM) without compromising binding curve quality or precision in estimates of *K_D_*. This approach expands the flexibility of FA assay design, enabling experiments to remain within theoretically appropriate ligand-limiting regimes while minimizing reagent consumption. Importantly, including Tween 20 enhanced the signal reproducibility and improved curve fitting, particularly in challenging low-volume formats.

Our results were validated using both a well-characterized thrombin–aptamer system and a clinically relevant aptamer–protein pair (s10yh2–HSA). In both cases, the optimized FA conditions yielded reliable and accurate anisotropy signals, confirming the broader utility of this approach for translational and diagnostic applications.

Overall, this work establishes a practical and low-cost method for improving FA-based biomolecular interaction studies by incorporating optimized surfactant conditions. The findings provide valuable guidance for researchers developing sensitive and reproducible FA assays, particularly in high-throughput and resource-limited settings and may benefit a wide range of aptamer–target systems beyond those explored here.

## Data Availability

The data presented in the study are openly available in KU ScholarWorks at https://hdl.handle.net/1808/36192 (accessed on 30 September 2025).

## Abbreviations

The following abbreviations are used in this manuscript:

TR	Texas Red
FAM	Fluorescein
FA	Fluorescence Anisotropy
SEM	Standard Error of the Mean
